# Huxley on Physiology

**Published:** 1901-11

**Authors:** 


### Abstracts and Selections.

### Huxley on Physiology.

In the recently published Life and Letters of Huxley, the subject of the biography in one of his letters says: "I would urge that a thorough study of human physiology is in itself an education broader and more comprehensive than much that passes under that name. There is no side of the intellect which it does not call into play, no region of human knowledge into which its roots or its branches do not extend; like the Atlantic between the Old and the New worlds, its waves wash the shores of the two worlds of matter and mind; its tributary streams flow from both; through its waters, as yet unfurrowed by the keel of any Columbus, lies the road, if such there be, from the one to the other; far away from the Northwest Passage of speculation, in which so many brave souls have been frozen up."—*Dietetic and Hygiene Gazette,* October, 1901. —R.

